# PD-L1 Expression in Muscle-Invasive Urinary Bladder Urothelial Carcinoma According to Basal/Squamous-Like Phenotype

**DOI:** 10.3389/fonc.2020.527385

**Published:** 2020-12-07

**Authors:** Bohyun Kim, Cheol Lee, Young A. Kim, Kyung Chul Moon

**Affiliations:** ^1^ Department of Pathology, Seoul National University College of Medicine, Seoul, South Korea; ^2^ Department of Pathology, Seoul Metropolitan Government-Seoul National University Boramae Medical Center, Seoul, South Korea; ^3^ Kidney Research Institute, Medical Research Center, Seoul National University College of Medicine, Seoul, South Korea

**Keywords:** programmed cell death-ligand 1, muscle-invasive bladder cancer, basal/squamous-like, cytokeratin 5/6, cytokeratin 14, GATA3, FOXA1, immunohistochemistry

## Abstract

Urothelial carcinoma (UC) is the most common histologic type of urinary bladder cancer, and muscle-invasive UC shows aggressive behaviors. Programmed cell death-1 (PD-1)/programmed cell death-ligand 1 (PD-L1) blockades have been approved as standard treatments for patients with advanced stage UC. A total of 166 muscle-invasive urinary bladder cancer (MIBC) patients, who underwent transurethral resection of the bladder or cystectomy from 2004 to 2010 were included. We evaluated PD-L1 expression by the SP142 and SP263 assays and classified the cases “positive” or “negative” according to the manufacturer’s recommendations. We performed immunohistochemistry (IHC) for cytokeratin (CK) 5/6, CK14, GATA3, FOXA1, and CK20 and classified samples as Basal-Squamous-like (BASQ) or non-BASQ subtype. The overall concordance rate for PD-L1 expression is 91.6% (152/166) (kappa = 0.732). The SP142 assay showed 15.1% positivity; the SP263 assay showed 23.5%. The high positivity in the SP142 and SP263 assay was significantly correlated with positive CK5/6, CK14 expression, negative GATA3, FOXA1, and CK20 expression. Classification according to IHC expression resulted in 12.0% (20/166) of samples being classified as BASQ subtype and 88.0% (146/166) of samples being classified as non-BASQ subtype. High positivity in the SP142 and SP263 assay was significantly correlated with the BASQ subtype (p < 0.001, both). Our study is the first to analyze the association of immunohistochemically defined BASQ and non-BASQ subtypes with two PD-L1 assays in MIBC. In conclusion, we revealed that a high PD-L1 positive rate in all PD-L1 assays was significantly associated with the BASQ-subtype, and these results suggest that the BASQ classification may be important to apply the PD-1/PD-L1 blockades in MIBC.

## Introduction

Urinary bladder cancer is the 10th most common cancer and 14th most common cause of cancer-related death worldwide (1). Urothelial carcinoma (UC) is the most common malignant urinary bladder tumor. Programmed cell death-1 (PD-1)/programmed cell death-ligand 1 (PD-L1) blockades have shown clinical treatment efficacy in patients with advanced or metastatic UC (2, 3). Atezolizumab was approved for locally advanced or metastatic UC, and the SP142 assay was approved as a companion diagnostic test. Durvalumab was approved for inoperable or metastatic disease, and the SP263 assay was approved as a complementary diagnostic test (4).

Immune checkpoint inhibitors have been a promising and effective treatment option for various malignant tumors (5–8). Blockade of the PD-1/PD-L1 signaling pathway has been the main focus of treatment strategies, and PD-L1 immunohistochemical expression has been shown to be involved in predicting immune checkpoint inhibitor therapy efficacy (9).

Various previous studies identified the gene expression profiles and molecular characteristics of UC (10–14). From these studies, UC can be classified into several intrinsic molecular subtypes. Each research group uses different subtype classifications, among which there are many similarities, and classification into a basal subtype and a luminal subtype is common. The basal subtype expresses high levels of KRT5, KRT6, KRT14, and CD44. The luminal subtype expresses high levels of UPKs, KRT20, GATA3, and FOXA1 (11, 12). Additionally, in a consensus meeting urinary bladder cancers with high expression of KRT5/6 and KRT14 and low expression of FOXA1 and GATA3 were designated as the Basal-Squamous-like (BASQ) subtype (15). In addition to these mRNA expression-based subtype classifications, a few studies have reported immunohistochemistry (IHC)-based subtype classifications (16, 17). In one study, the IHC expression of basal (KRT5/6) and luminal (GATA3) markers reportedly reflected the molecular subtype with high accuracy (16). In another study, the basal/squamous cell carcinoma-like group was reported to show a tumor phenotype with high KRT6 and KRT14 and low FOXA1 and GATA3 expression by IHC (17).

The IMvigor210 phase II trial and another study reported that in locally advanced UC patients, molecular subtypes showed different clinical prognoses and clinical responses to the PD-L1 inhibitor atezolizumab (18–20). TCGA molecular subtypes showed different tumor cell (TC) PD-L1 expression and immune cell (IC) PD-L1 expression and different responses to atezolizumab (18, 19). In addition, Hodgson et al. suggested distinguishing luminal and basal subtypes of muscle-invasive urinary bladder UC (MIBC) using cytokeratin (CK) 5/6 and GATA3 IHC, and after such classification, the basal subtype showed a significant association with the abundance of CD8+ T cell expression and with high PD-L1 positivity identified by the SP263 assay (21).

Based on these findings, in this study, we classified MIBC into either BASQ or non-BASQ subtypes according to the expression profile of the CK5/6, CK14, GATA3, and FOXA1 and investigated PD-L1 expression in MIBC in BASQ and non-BASQ subtypes.

## Materials and Methods

### Patients and Tissue Samples

In total, MIBC tissues were from 163 patients who underwent transurethral resection of the bladder and 42 patients who underwent cystectomy. 104 patients were retrieved from Seoul National University Hospital (SNUH), 101 patients from Seoul Metropolitan government-Seoul National University Boramae Medical Center between 2004 and 2010. Patients without prior history of preoperative treatment were selected, and only 166 pure UCs showing no specific differentiation or variant histology were included in the study. Clinicopathologic data, including histologic subtype, were collected by reviewing medical records. We reviewed hematoxylin and eosin (H&E)-stained slides to confirm the diagnosis and identify various pathologic parameters.

A tissue microarray (TMA) block was prepared from formalin-fixed paraffin-embedded tissue blocks (SuperBio-Chips Laboratories, Seoul, South Korea). Two cores (2 mm in diameter) containing the invasive tumor area were obtained from each patient.

This study was approved by the Institutional Review Board (IRB) of SNUH (IRB No H-1909-033-1062) and was performed in accordance with the principles of the Declaration of Helsinki. Informed consent was waived by the IRB.

### Immunohistochemical Staining

For immunohistochemical analyses, the TMA blocks were cut at 4 µm thickness. All PD-L1 immunohistochemical staining was carried out on a Benchmark Ultra System (Ventana Medical Systems, Tucson, AZ). Clone SP142 (Ventana Medical Systems; retrieval: CC1 48′; incubation: 16′; RTU dilution) and clone SP263 [Ventana Medical Systems; retrieval: CC1 40′; incubation: 32′; ready to use (RTU) dilution] were used in accordance with the manufacturer’s guidelines. The SP142 assay used tonsil tissue, and the SP263 assay used placenta tissue as control tissue. Two control tissues were used for each staining run, one as a positive control using an antibody reagent and one as a negative control using a negative reagent.

Additional immunohistochemical staining for CK5/6, CK14, GATA3, FOXA1, and CK20 was performed using a Ventana Benchmark XT automated staining system (Ventana Medical Systems). Mouse monoclonal antibodies against CK5/6 (1:100; D5/16 B4; Dako, Glostrup, Denmark), CK14 (1:300; LL002; Cell Marque, Rocklin, CA), GATA3 (1:500; L50-823; Cell Marque), FOXA1 (1:500; PA5-27157; Thermo Fisher, Waltham, MA), and CK20 (1:50; Ks 20.8; Dako) were used.

### Immunohistochemical Scoring

PD-L1 expression of TC was evaluated based on the proportion of tumor cells exhibiting membranous staining of any intensity. PD-L1 expression of IC was evaluated based on the proportion of tumor-associated immune cells with membranous, cytoplasmic, or punctate staining at any intensity and the proportion of tumor area that was occupied by PD-L1 staining IC of any intensity. Each PD-L1 expression type was dichotomized as “positive” or “negative” according to the manufacturer’s recommendations.

Cut-off criteria for PD-L1 expression positivity are summarized in **Table 1**. For CK5/6, CK14, and CK20, >20% expression was defined as positive expression status. 20% cut-off value was reported in previous studies to be ideal for classification of molecular subtypes in bladder cancer (16, 21). So, we also applied the same criteria. GATA3 and FOXA1 were based on nuclear staining, and percentage of stained cells and staining intensity were also considered. Staining intensity was scored 0 to 3+ (0: no staining, 1+: weak staining, 2+: moderate staining, 3+: strong staining). 3+ staining intensity on more than 20% of tumor cells was defined as positive expression. The average of the two core values was evaluated as the final result.

**Table 1:** Positive criteria of PD-L1 assays.

	Cutoff for positivity
PD-L1 (SP142)	Presence of discernible PD-L1 staining of any intensity in tumor-infiltrating immune cells covering ≥ 5% of tumor area occupied by tumor cells, associated intratumoral, and contiguous peritumoral stroma
PD-L1 (SP263)	≥25% of tumor cells exhibit membrane staining; or, ICP > 1% and IC+ ≥ 25%; or, ICP = 1% and IC+ = 100%

IC+, Percentage of tumor-associated immune cells with staining; ICP, Percent of tumor area occupied by any tumor-associated immune cells.

We classified all cases as either BASQ or non-BASQ based on CK5/6, CK14, GATA3, and FOXA1 IHC expression. The BASQ subtype included cases with both positive CK5/6 and CK14 expression and both negative GATA3 and FOXA1 expression. The remaining cases were classified as non-BASQ subtype.

Two pathologists (BK and CL) evaluated the IHC staining at two different time points, without awareness of previous results at the second evaluation. The discrepant cases were evaluated by another pathologist (KM) for the final consensus results.

### Statistical Analysis

The concordance rate of PD-L1 expression between the SP142 assay and the SP263 assay was evaluated. Cohen’s kappa coefficient of agreement was calculated: the level of concordance could be classified as poor (kappa = 0.00), slight (kappa = 0.00–0.20), fair (kappa = 0.21–0.40), moderate (kappa = 0.41–0.60), substantial (kappa = 0.61–0.80) or almost perfect (kappa = 0.81–1.00) (22). The association between PD-L1 expression and immunohistochemical results was evaluated by the chi-square test or Fisher’s exact test. Statistical analyses were performed using SPSS software (version 23; IBM, Armonk, NY, USA). Two-sided p values of <0.05 were considered to be statistically significant.

## Results

### Clinicopathologic Characteristics of Patients

Overall, 166 patients were included in this study, including 139 males and 27 females. The age at the time of diagnosis ranged from 37 to 87 years, with a mean age of 76 years. According to the 8th edition of the TNM staging system of the AJCC, 150 patients were in pT2, 10 patients were in pT3, and six patients in pT4. According to the WHO/ISUP grading system, seven cases were classified as low grade, and 159 cases as high grade. The association of BASQ classification with clinicopathologic characteristics was summarized in **Table 2**. The BASQ subtype was not correlated with old age (>69 years), gender, high WHO/ISUP grade, and high T category.

**Table 2:** Clinicopathologic characteristics of patients and association with BASQ classification.

	Non-BASQ N(%)	BASQ N(%)	Total	p value
Age (years)				
≤69	62 (42.5%)	12 (60.0%)	74	0.139
>69	84 (57.5%)	8 (40.0%)	92	
Gender				
Male	121 (82.9%)	18 (90.0%)	139	0.418*
Female	25 (17.1%)	2 (10.0%)	27	
Nuclear grade				
Low	6 (4.1%)	1 (5.0%)	7	0.853*
High	140 (95.9%)	19 (95.0%)	159	
T category				
T 2	132 (90.4%)	18 (90.0%)	150	0.953*
T 3–4	14 (9.6%)	2 (10.0%)	16	

*Fisher’s exact test.

BASQ, Basal-Squamous-like tumors.

### PD-L1 Assays

The results of the SP142 and SP263 assays are summarized in **Table 3**. The overall concordance rate for PD-L1 expression between SP 142 and SP 263 was 91.6% (152/166) (kappa = 0.732). The SP142 assay showed 15.1% (25/166) positivity at the IC 5% cut-off. The mean percentage of IC expression was 2.7% (range, 0–80), and the mean percentage of TC expression was 3.1% (range, 0–80). The SP263 assay showed 23.5% (39/166) positivity at the TC or IC 25% cut-off. When further subdivided, 11 cases met only the TC criteria, 10 cases met only the IC criteria, and 18 cases met both TC and IC criteria. The mean percentage of IC expression was 9.7% (range, 0–80), and the mean percentage of TC expression was 11.3% (range, 0–95). The union with either SP142 or SP263 assays of positive cases was 39 cases. The PD-L1 IHC expression values of the 39 positive results cases are summarized in **Table 4**. Among them, all 25 cases positive for the SP142 assay were also positive for the SP 263 assay, and 14 cases were positive for SP263 assay only. The SP263 assay showed higher PD-L1 expression than the SP142 assay in both tumor cells and immune cells.

**Table 3:** Distribution of PD-L1 expression in MIBC.

PD-L1 assay	Positive cell	Positive rate					
		0%	1–4%	5–9%	10–24%	25–49%	50–100%
SP142	TC	119 (71.7%)	21 (12.7%)	9 (5.4%)	11 (6.6%)	4 (2.4%)	2 (1.2%)
	IC	88 (53.0%)	53 (32.0%)	11 (6.6%)	10 (6.0%)	2 (1.2%)	2 (1.2%)
SP263	TC	99 (59.6%)	22 (13.3%)	8 (4.8%)	8 (4.8%)	10 (6.0%)	19 (11.5%)
	IC	58 (34.9%)	41 (24.7%)	20 (12.0%)	19 (11.5%)	19 (11.5%)	9 (5.4%)

MIBC, Muscle-invasive urinary bladder urothelial cell carcinoma; TC, tumor cell; IC, immune cell.

**Table 4:** PD-L1 assays values of the 39 positive results cases.

Positive cases No.	SP142			SP263		
	IC positive(%)	TC positive(%)	Result	IC positive(%)	TC positive(%)	Result
1	0	1	Neg	30	5	Pos
2	0	3	Neg	10	57.5	Pos
3	0	5	Neg	5	30	Pos
4	0.5	0	Neg	0	80	Pos
5	0.5	0	Neg	25	5	Pos
6	1	0	Neg	6	75	Pos
7	1	0	Neg	25	5	Pos
8	1	0	Neg	27.5	25	Pos
9	1	0	Neg	30	35	Pos
10	1	0	Neg	40	1	Pos
11	1	0	Neg	40	5	Pos
12	1	1	Neg	15	75	Pos
13	2	0	Neg	27.5	5	Pos
14	2.5	0.5	Neg	1	77.5	Pos
15	5	0	Pos	0	25	Pos
16	5	0	Pos	25	25	Pos
17	5	1	Pos	25	1	Pos
18	5	1	Pos	25	22.5	Pos
19	5	1	Pos	30	25	Pos
20	5	5	Pos	7.5	90	Pos
21	5	15	Pos	30	80	Pos
22	6	12.5	Pos	10	85	Pos
23	7.5	20	Pos	5	95	Pos
24	7.5	25	Pos	30	0	Pos
25	7.5	35	Pos	72.5	75	Pos
26	10	1	Pos	10	50	Pos
27	10	1	Pos	45	82.5	Pos
28	10	5	Pos	40	27.5	Pos
29	10	5	Pos	42.5	30	Pos
30	10	5	Pos	75	70	Pos
31	10	10	Pos	40	50	Pos
32	15	1	Pos	80	20	Pos
33	15	5	Pos	57.5	60	Pos
34	17.5	10	Pos	70	55	Pos
35	17.5	20	Pos	30	80	Pos
36	20	20	Pos	55	40	Pos
37	30	25	Pos	50	42.5	Pos
38	55	75	Pos	55	50	Pos
39	80	80	Pos	80	92.5	Pos

IC, immune cell; Neg, negative; No., number; Pos, positive; TC, tumor cell.

Comparisons of PD-L1 IHC expression with the SP142 and SP263 assays between positive and negative groups classified according to CK5/6, CK14, GATA3, FOXA1 and CK20 expression levels are summarized in **Table 5**. High positivity in both the SP142 and SP263 assays was significantly correlated with positive CK5/6, positive CK14, negative GATA3, negative FOXA1 and negative CK20 expression.

**Table 5:** Relationship between PD-L1 positivity and CK5/6, CK14, GATA3, FOXA1, and CK20 expression.

		SP142			SP263		
		Neg (n = 80)	Pos (n = 24)	p	Neg (n = 75)	Pos (n = 28)	p
CK5/6	Neg	102 (72.3%)	5 (20.0%)	<0.001	99 (78.0%)	8 (20.5%)	<0.001
	Pos	39 (27.7%)	20 (80.0%)		28 (22.0%)	31 (79.5%)	
CK14	Neg	129 (91.5%)	15 (60.0%)	<0.001*	121 (95.3%)	23 (59.0%)	<0.001
	Pos	12 (8.5%)	10 (40.0%)		6 (4.7%)	16 (41.0%)	
GATA3	Neg	55 (39.0%)	19 (76.0%)	0.001	44 (34.6%)	30 (76.9%)	<0.001
	Pos	86 (61.0%)	6 (24.0%)		83 (65.4%)	9 (23.1%)	
FOXA1	Neg	81 (57.4%)	21 (84.0%)	0.012	69 (54.3%)	33 (84.6%)	0.001
	Pos	60 (42.6%)	4 (16.0%)		58 (45.7%)	6 (15.4%)	
CK20	Neg	67 (47.5%)	22 (88.0%)	<0.001	55 (43.3%)	34 (87.2%)	<0.001
	Pos	74 (52.5%)	3 (12.0%)		72 (56.7%)	5 (12.8%)	

*Fisher’s exact test.

CK, cytokeratin; Neg, negative; Pos, positive.

### PD-L1 Expression in BASQ or Non-BASQ Subtype

**Figure 1:**
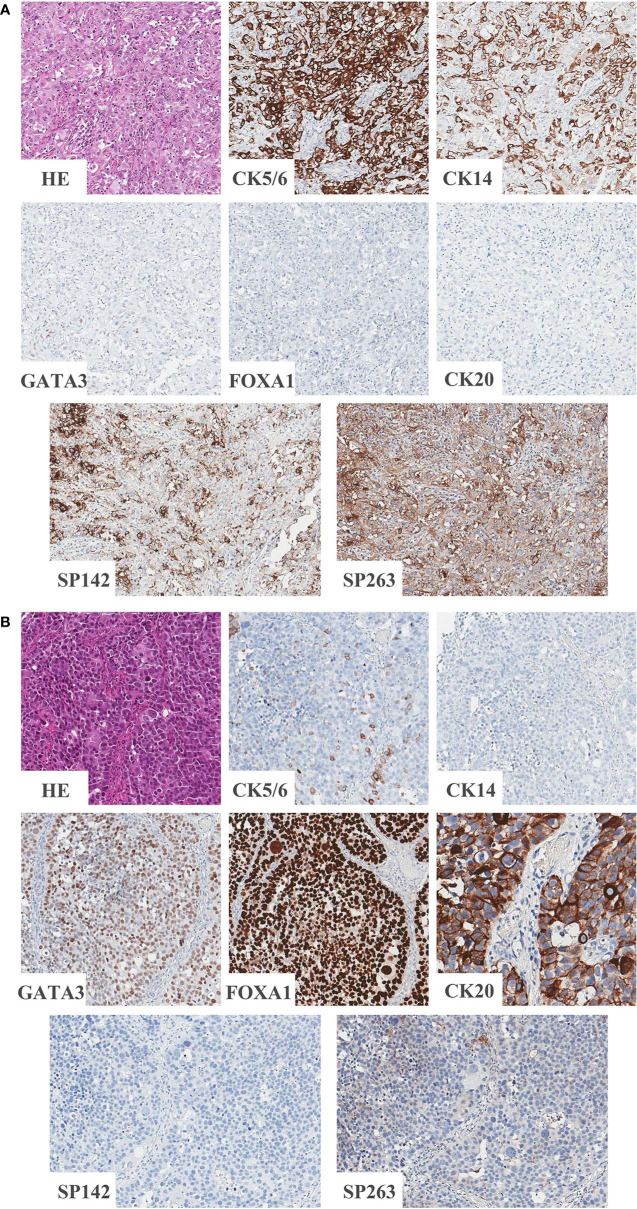
Representative images of BASQ and non-BASQ subtype. **(A)** BASQ subtype is positive for CK5/6, CK14 and negative for GATA3, FOXA1, CK20. This subtype is highly correlated with positivity of PD-L1 assays (SP142 and SP263). **(B)** Non-BASQ subtype is negative for CK5/6, CK14 and positive for GATA3, FOXA1, CK20. PD-L1 assays (SP142, SP263) are negative.

Classification according to CK5/6, CK14, GATA3 and FOXA1 IHC expression resulted in 12.0% (20/166) of samples being classified as the BASQ-subtype and 88.0% (146/166) of samples being classified as the non-BASQ subtype. High positivity in both the SP142 and SP263 assays was significantly correlated with the BASQ subtype (p < 0.001, both) (**Figure 1** and **Table 6**).

**Table 6:** Comparison of PD-L1 positivity and BASQ phenotype.

	SP142			SP263		
	Neg(n = 80)	Pos(n = 24)	p	Neg(n = 75)	Pos(n = 28)	p
Non-BASQ	131 (92.9%)	15 (60.0%)	<0.001*	123 (96.9%)	23 (59.0%)	<0.001*
BASQ	10 (7.1%)	10 (40.0%)		4 (3.1%)	16 (41.0%)	

*Fisher’s exact test.

BASQ, Basal-Squamous-like; Neg, negative; Pos, positive.

## Discussion

Great progress has been reported in research on the molecular classification of UC. Molecular classification of bladder cancer based on mRNA expression has been suggested by The Cancer Genome Atlas (10), the MD Anderson group (11), the University of North Carolina group (12), and the Lund Bladder Cancer Research group (17). Each group suggested different classifications, but the most basic classification scheme includes classification into basal and luminal subtypes. Additionally, IHC based classifications that reflect mRNA expression and are also easy and accessible in practical settings have been suggested. Dadhania et al. suggested IHC positivity for CK 5/6 and CK14 as basal subtype markers and IHC positivity of GATA3, CK20, and uroplakin as luminal subtype markers and suggested CK5/6 and GATA3 to be representative IHC markers (16). In addition, Choi et al. suggested a BASQ subtype with the immunohistochemical expression pattern of CK5/6 (+), CK14 (+), GATA3 (−) and FOXA1 (−) (11).

Different molecular subtypes have been reported to have different prognoses and chemotherapy sensitivities. Additionally in phase II clinical trial of CheckMate, 275 different Nivolumab immunotherapy responses were reported among molecular subtypes in patients with metastatic UC, of which it was highest in the TCGA cluster III (23). Additionally, in a clinical trial using the SP142 assay, mRNA based TCGA clusters III and IV, which correspond to the basal type, showed highly enriched PD-L1 IC and TC expression, and TCGA cluster II, which corresponds to the luminal type, showed a significantly higher response to atezolizumab, a humanized monoclonal anti PD-L1 antibody, than clusters III and IV (18). Our present results showing high PD-L1 expression in BASQ subtype are in line with previous research.

In a previous study, Hodgson et al. suggested that MIBC could be classified into luminal and basal subtypes using CK5/6 and GATA3 IHC, and each subtype was analyzed for its PD-L1 SP263 expression and tumor infiltrating lymphocytes. The results showed that PD-L1 positivity was more common in the basal subtype and that CD8+ T cells and PD-1 expression were also significantly associated with the basal subtype (21).

In this study, we additionally analyzed the PD-L1 expression status using SP142 as well as SP263 in MIBC immunohistochemically defined molecular subtypes. We performed IHC with five antibodies to classify the BASQ subtype which was defined at a previous consensus meeting. We designated cases with positive CK5/6 and CK14 IHC expression and negative GATA3 and FOXA1 IHC expression as BASQ subtype. We evaluated PD-L1 expression using two PD-L1 assays (SP142 and SP263), and the overall concordance rate of PD-L1 positive status was substantial, in line with previous studies (24). We demonstrated that a high PD-L1 positive rate was significantly associated with the BASQ subtype. Furthermore, we demonstrated that a high PD-L1 positive rate was significantly associated with positive CK5/6 and CK14 expression and negative GATA3, FOXA1 and CK20 expression.

T lymphocytes eliminate tumor cells through immune surveillance using T cell receptor and major histocompatibility complex interaction. The interaction of PD-1 expressed in T lymphocyte with PD-L1 expressed in tumor cell leads to obstacles in immune regulation described above. PD-1/PD-L1 pathway is used by tumor cells as a method of immune evasion. But, PD-L1 expression on clinical prognosis is poorly defined in UC. Some studies have suggested that PD-L1 overexpression was correlated with poor prognosis in bladder cancer (25–27). On the other hand, some studies reported that PD-L1 expression has no correlation with clinical outcome in UC (28, 29).

The role of PD-L1 expression as a predictive biomarker also has many points to consider. There are clinical trial results that show that PD-L1 expression and response rate of PD-1/PD-L1 blockades are insufficiently related (30). However, currently, it is clear that PD-L1 expression is a companion or complementary diagnostic test of PD-1/PD-L1 blockades and a potential predictive biomarker. And it is generally accepted that PD-1/PD-L1 blockades has been known to have a higher efficacy in patients whose immune cells express PD-L1. The positive PD-L1 expression rate in BASQ and non-BASQ subtype UC can help to determine a treatment strategy in MIBC. Classification of MIBC according to the BASQ/non-BASQ classification could be suggested as a potential marker for PD-1/PD-L1 blockade treatment.

There are several limitations to this study. First, PD-L1 expression may show intratumoral heterogeneity. To compensate for this deficiency, two different tumor cores were used for each case, but there is a possibility that this result may not represent the overall tumor expression rate. Next, the cut-off value of the CK5/6, CK14, GATA3, and FOXA1 expression for BASQ classification has not been clearly established. For accurate analysis, we investigated various previous studies, identified cut-off values that were proved appropriate, and applied them to our study.

In conclusion, we demonstrated that BASQ subtype is highly associated with the positivity of the PD-L1 assays. To the best of our knowledge, our study is the first to analyze the association of immunohistochemically defined BASQ or non-BASQ subtype with two PD-L1 assays (SP142 and SP263) in MIBC. The significant difference of positive rate of PD-L1 assays according to BASQ classification suggests that BASQ classification may be important to apply the PD-1/PD-L1 blockade treatment in patients with MIBC.

## Data Availability Statement

The datasets generated for this study are available on request to the corresponding author.

## Ethics Statement

The studies involving human participants were reviewed and approved by Institutional Review Board (IRB) of Seoul National University Hospital. Written informed consent for participation was not required for this study in accordance with the national legislation and the institutional requirements.

## Author Contributions

CL and KM designed and performed the experiments. BK, CL, and YK analyzed the data. BK wrote the manuscript. KM supervised the entire process. All authors contributed to the article and approved the submitted version.

## Funding

This research was supported by Basic Science Research Program through the National Research Foundation of Korea (NRF) funded by the Ministry of Education (2018R1D1A1B07045763).

## Conflict of Interest

The authors declare that the research was conducted in the absence of any commercial or financial relationships that could be construed as a potential conflict of interest.
